# SP70 is a potential biomarker to identify gastric fundic gland neoplasms

**DOI:** 10.1186/s12957-022-02564-8

**Published:** 2022-04-25

**Authors:** Rongkui Luo, Wen Huang, Lingli Chen, Yalan Liu, Lei Xu, Xiaolei Zhang, Chen Xu, Yingyong Hou

**Affiliations:** Department of Pathology, Zhongshan Hospital, Fudan University, No. 180 Fenglin Rd, Xuhui District, Shanghai, 200032 People’s Republic of China

**Keywords:** Fundic gland polyps, Oxyntic gland adenomas, Gastric adenocarcinomas of fundic gland type, SP70

## Abstract

**Background:**

Gastric neoplasms with fundic gland differentiation include oxyntic gland adenomas (OGAs) and gastric adenocarcinomas of fundic gland type (GA-FGs). Due to their well-differentiated and similar morphology with normal fundic glands, it is usually challenging to identify these lesions in pathological diagnosis, especially in biopsy specimens. This study aims to explore and verify the potential role of a newly developed monoclonal antibody (McAb) NJ001 (SP70) in differentiating fundic neoplasms from non-neoplastic fundic gland lesions.

**Methods:**

Twenty-three cases of histological confirmed gastric fundic gland neoplasms were obtained, including 12 cases of OGAs and 11 of GA-FGs. Fifty cases of fundic gland polyps (FGPs) were taken as the control group. Six cases of well-differentiated gastric neuroendocrine tumors (NETs) (easily misdiagnosed) were also obtained. Key clinicopathological information was collected. SP70 immunostaining was performed (with para-tumor normal fundic glands as internal control). The positive intensity and staining pattern of SP70 were analyzed and compared.

**Results:**

In normal gastric mucosa, SP70 was strongly and diffusely stained on the cytoplasm in fundic glands, but not in the foveolar epithelium. Therefore, a zonal distribution of SP70 was observed in normal mucosa. FGPs (50/50, 100%) shared a similar expression pattern with normal fundic glands. In fundic gland neoplasms, a significant down-expression of SP70 was observed in both OGAs and GA-FGs. The positive rate of SP70 in fundic gland neoplasms (6/23, 26.1%) was significantly lower than that in FGPs (100%) (*P<0.0001*). There was no difference in SP70 expression between OGAs (3/12, 25.0%) and GA-FGs (3/11, 27.2%) group (*P*>0.05). In these 6 NET cases, SP70 was weak to moderate intensity in the majority of tumor cells (with a different expression pattern).

**Conclusion:**

Down-expression of SP70 is a specific feature to fundic gland neoplasms including OGAs and GA-FGs. Therefore, SP70 can serve as a potential biomarker in the identification and differential diagnosis of fundic gland neoplasms.

## Introduction

Gastric neoplasm with differentiation toward the fundic gland is a novel entity [1]. First discovered by Ueyama et al. [2] in 2010, gastric adenocarcinoma of fundic gland type (GA-FG) was proposed as a rare neoplastic lesion (with the ability to invade the stroma), mainly composed of chief cells. Distinct from other gastric carcinomas, GA-FG is identified to have a low-grade malignancy (with rare perineural or lymphovascular invasion) and a good prognosis [3]. Two years later, from a prognostic perspective, Singhi et al. [4] suggested that the terminology of oxyntic gland polyp/adenoma (OGA) was more suitable for the lesions limited within the mucosa. This subtype shows the features of slow progression and a rather benign clinical course [5]. Currently, both OGA and GA-FG have been adopted by the World Health Organization’s (WHO) classification of digestive system tumors (5th edition) [6].

Due to its well histopathological differentiation, the morphological characters of gastric fundic gland neoplasms may resemble to that of normal fundic glands [7]. Despite the architectural abnormities, the minimal cellular atypia makes the diagnosis of these neoplasms more challenging and may lead to misdiagnosis (such as FGPs), especially in biopsy specimens [3]. It is important clinically to distinguish neoplastic lesions (OGA and GA-FG) from the non-neoplastic lesions, for the latter may not require a clinical intervention [8, 9]. Previous studies indicated that sporadic FGPs were self-limiting neoplasms, which had little chance of transforming into carcinomas [4], and the patients only needed to undergo routine follow-up. However, neoplasms with fundic gland differentiation are considered to share similar histogenesis [4], that is frequent submucosa invasion. Therefore, further clinical intervention including endoscopic resection or even complete surgical excision [10] is appropriate for such neoplasms, in case of further progression [11]. NET is the other disease that requires differentiation from OGA, for some of them share similar architectural patterns and biomarker expression (synaptophysin and/or CD56 positive) [1]. A panel of immunohistochemical markers has been used as the auxiliary methodology to generate a precise diagnosis, when a diagnosis could not be made by routine H&E staining. The current markers include MUC5AC, MUC6, Pepsinogen I, and H+/K+-ATPase, and these markers are very helpful to verify fundic glands differentiation [12]. Nevertheless, because normal fundic glands or non-neoplastic lesions of fundic glands (FGPs) harbor the same IHC phenotype as well, it is unlikely to make the diagnosis only based on IHC findings. Therefore, these markers can only be used as support data. Newly developed specific IHC biomarkers that can distinguish fundic gland neoplasms from non-neoplastic lesions are needed to improve the diagnostic difficulties, especially in cases with atypical morphology and biopsy specimens with limited tissue.

SP70 is an antigen of non-small-cell lung cancer (NSCLC) specifically identified by the McAb NJ001 [13, 14]. It has been produced by immunizing mice with human SPC-A1 lung adenocarcinoma live-cell antigen [14]. In this study, the expression pattern of SP70 in a series of fundic gland lesions was studied. It showed that the down-expression of SP70 was a characteristic feature of neoplastic lesions, and this biomarker may serve as a diagnostic marker for such a rare entity.

## Materials and methods

### Patients

Archival paraffin blocks of 23 cases of fundic gland neoplasms were obtained from Zhongshan Hospital, Fudan University (Shanghai, China), from Jan. 2017 to Dec. 2020, among which 12 cases were OGAs and 11 were GA-FGs. The adjacent normal fundic glands served as an internal control. Six cases of well-differentiated NETs (as the main differential diagnosis) located in the gastric body were obtained. Fifty cases of FGPs were taken as the control group. The study protocol and the comprehensive written informed consent were assigned.

### Immunohistochemistry

Paraffin-embedded specimens were sectioned and were subjected to immunohistochemistry. Monoclonal antibodies were mouse mAb NJ001 (1:400; NM001-1; Code Biotech, Jiangsu, China). Besides, the markers for chief cell and parietal cell differentiation were used, including Pepsinogen-I (1:400; 7G3; Abcam, Shanghai, China), MUC 6 (1:100; MRQ-20; Gene Tech, Shanghai, China), MUC 5AC (1:400; 45M1; Gene Tech, Shanghai, China), and H+/K+-ATPase (1:400; poly; Jiehao Biotechnology, Shanghai, China).

SP70 was mainly localized in the cellular cytoplasm. Five hundred cells were assessed, and the percentage of SP70 positive cells was counted. The result was assigned as negative when the SP70 positive cells were <5% and ≥5% as positive.

### Histopathological assessment

Histological features, immunohistochemical staining, and clinicopathological data were assessed. Histopathological reviews and immunohistochemical analysis were performed by three pathologists. Diagnostic criterion takes reference from the 5th edition of WHO classification of gastrointestinal tract tumors (2019) [6].

### Statistical analysis

SPSS statistics 21.0 was used to carry out statistical analyses. The statistical differences between different groups were compared by the *χ*^2^ test. The *P*-value <0.05 was significant.

## Results

### Clinicopathological data

Histopathological and clinical findings were presented in Table 1 (with 12 cases of OGAs and 11 cases of GA-FGs). The patients ranged in age from 37 to 80 years old (median, 63 years old), with a male-to-female ratio of 14: 9. The lesions were located in fundus ventriculi (*n*=9) and corpus ventriculi (*n*=14) and measured 0.3–2.0 cm in diameter.

**Table 1:** The clinical and histopathological features of 23 cases

Case sex	Age (years)	Group #	Ki67	SP70	Macroscopic type	Location	Size (mm)	Treatment	Pigment (/HPF)	Mitosis	Cellular atypia	Invasion depth	Vascular invasion	Ulcer	Follow up
1. M	38	A	1%	+	Protruding	Fundus	6	Biopsy+polypectomy	Absent	0	Mild	Mu	Absent	Absent	NED
2. F	57	A	2%	+	Protruding	Corpus	6	Polypectomy	Absent	0	Mild	Mu	Absent	Absent	NED
3. F	54	A	5%	−	Protruding	Corpus	6	Polypectomy	Absent	0	Mild	Mu	Absent	Absent	NED
4. F	59	unclassified	5%	−	Superficial elevated	Corpus	7	Surgery	Absent	1	Moderate	Mu	Absent	Absent	NED
5. F	80	A	2%	−	Protruding	Fundus	6	Polypectomy	Absent	0	Mild	Mu	Absent	Absent	NED
6. M	64	A	5%	−	Protruding	Corpus	5	Biopsy+ESD	Absent	0	Mild	Mu	Absent	Absent	NED
7. F	67	A	5%	−	Protruding	Corpus	5	EMR	Absent	0	Mild	Mu	Absent	Absent	NED
8. M	71	A	2%	−	Superficial elevated	Corpus	15	ESD	Absent	0	Mild	Mu	Absent	Absent	NED
9. M	68	A	5%	−	Superficial flat	Corpus	5	ESD	Absent	0	Mild	Mu	Absent	Absent	NED
10. M	51	unclassified	5%	−	Superficial elevated	Fundus	15	Proximal gastrectomy	Absent	1	Moderate	Mu	Absent	Absent	NED
11. F	66	A	2%	−	Protruding	Corpus	6	Biopsy	Absent	0	Mild	Mu	Absent	Absent	NED
12. M	73	A	5%	+	Protruding	Corpus	10	ESD	Absent	0	Mild	Mu	Absent	Absent	NED
13. M	60	B	10%	+	Protruding	Corpus	10	ESD	Absent	0	Mild	SM1(110μm)	Absent	Absent	NED
14. M	37	C	1%	−	Superficial flat	Corpus	8	ESD	Absent	1	Mild to moderate	SM1(363μm)	Present	Absent	NED
15. M	71	C	8%	+	Protruding	Fundus	20	EMR	Absent	2	Moderate	SM1(134μm)	Absent	Absent	NED
16. F	54	B	5%	+	Submucosal tumor	Fundus	5	Proximal gastrectomy	Absent	0	Mild	SM1(235μm)	Absent	Absent	NED
17. M	56	C	60%	−	Submucosal tumor	Fundus	15	ESD+total gastrectomy	Present	1	Marked	SM2(597μm)	Absent	Absent	NED
18. M	63	B	10%	−	Protruding	Fundus	5	ESD	Absent	0	Mild	SM1(100μm)	Absent	Absent	NED
19. M	45	B	10%	−	Superficial elevated	Fundus	6	ESD	Absent	0	Mild	SM1(159μm)	Absent	Absent	NED
20. M	55	B	2%	−	Superficial elevated	Corpus	15	ESD	Present	0	Mild	SM1(150μm)	Absent	Absent	NED
21. F	74	B	2%	−	Superficial elevated	Corpus	6	ESD	Absent	0	Mild	SM1(100μm)	Absent	Absent	NED
22. F	60	B	2%	−	Superficial elevated	Fundus	8	ESD	Absent	0	Mild to moderate	SM2(1500μm)	Absent	Absent	NED
23. M	71	C	15%	−	Infiltrative ulcerative	Corpus	20	Biopsy+total gastrectomy	Absent	4	Marked	Subserosa	Present	Present	12*

*M* male, *F* female, *Mu* mucosal, *SM* submocosal, *ESD* endoscopic submucosal dissection, *EMR* endoscopic mucosal resection, *HPF* per high-power field, *NED* no evidence of disease. *Lost to follow-up after 12 months. ^#^Grouped by Tetsuo Ushiku’s research mentioned in reference [5]. Group A: intramucosal tumor with typical histologic features; Group B: submucosal invasive tumor with typical histologic features; Group C: submucosal invasive tumor with atypical histologic features

### Endoscopic findings

**Fig. 1:**
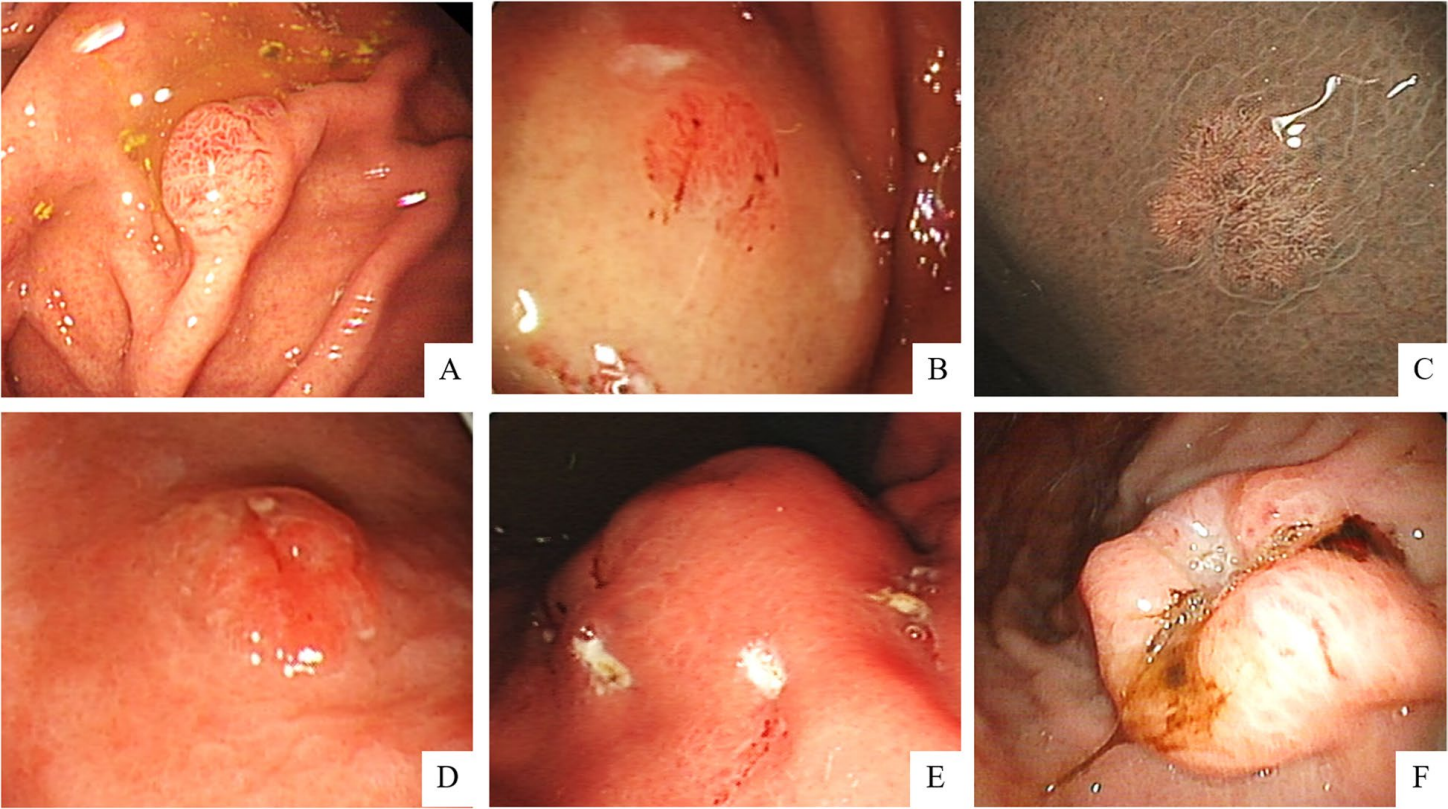
Representative endoscopic imaging from the above 23 cases. White light endoscopy revealed. **A** A protruding lesion. **B**, **C** Superficial flat type. **D** Tumor with superficial elevated appearance. **E** Submucosal tumor (SMT)-like shape was noted. **F** An infiltrative ulcerative tumor

Endoscopic findings showed that 47.8% (11/23) tumors had a protruding type (Fig. 1A), the superficial flat shape was noted in 34.8% (8/23) cases (Fig. 1B, C), 1 (1/23, 4.3%) case had a superficial elevated appearance (Fig. 1D), the submucosal tumor (SMT)-like shape was noted in 8.7% (2/23) tumors (Fig. 1E), and only 1 (1/23, 4.3%) case was recognized by an infiltrative ulcerative appearance (Fig. 1F).

### Histopathological analysis

**Fig. 2:**
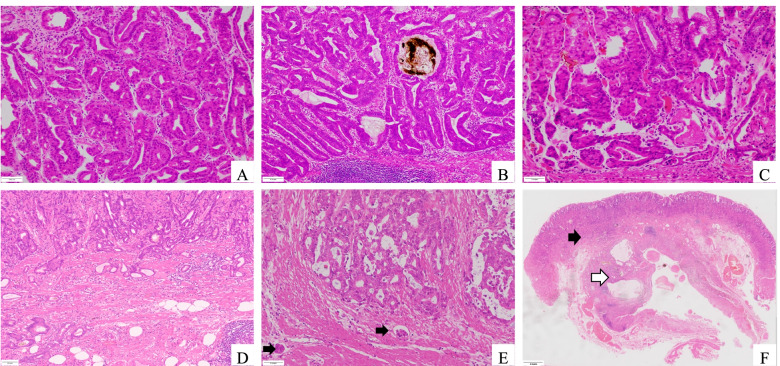
Histopathological features of fundic gland neoplasm. **A** Oxyntic gland adenoma (OGA) always consists of clustered and irregular fundic glands (HE, x100). **B** Chief cell–predominant OGAs with scattered parietal cells. Pigments could be observed in the dilated glands. **C** Complex glands with mild atypia neoplastic cells. **D** Gastric adenocarcinoma of fundic gland type (GA-FG) with submucosal invasion. **E** Muscular infiltration and vascular invasion (as indicated by the arrows) could be observed. **F** GA-FG (black arrow) with gastritis cystica profunda (white arrow)

Histologically, all lesions of OGAs were located in the mucosa layer (Fig. 2A, C). Pigments could be observed in 2 cases of OGAs (Fig. 2B). As for the GA-FGs, 10 of the tumors extended into the submucosa with an infiltrative pattern (Fig. 2D, E) and 1 case showed invasion into the sub-serosa.

According to Tetsuo et al. [5], we attempted to classify all the cases into 3 groups. Based on the criteria (histologic assessment), the patients were classified as group A (intramucosal tumor) in 11 cases, group B (submucosal invasive tumor with typical histologic features) in 7 cases, and group C (submucosal invasive tumor with atypical histologic features) in 4. Two patients could not be clearly classified according to the present criteria (shown in Table 1).

Intravascular cancer emboli could be observed in 2 patients (cases 14 and 23, in Table 1). Case 23 was lost in the follow-up with a final follow-up time of 12.5 months (operative time as a starting point, to patient final visiting for the end), and no recurrence or metastasis was found in the rest 22 patients (followed up for 10–49 months).

### Expressions of fundic gland-associated markers

**Fig. 3:**
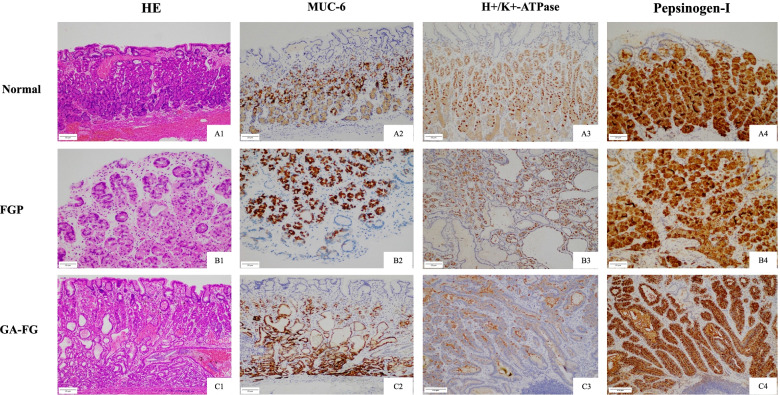
The expression of muc6, H+/K+-ATPase, and Pepsinogen-I in FGPs/normal fundic glands, OGPs, and GA-FGs

MUC6 presented a strong expression in fundic gland cells (in contrast with MUC5AC in foveolar epithelium) (Fig. 3A2, B2, C2). The markers for parietal cell (H+/K+-ATPase) (Fig. 3A3, B3, C3) and chief cell (pepsinogen-I) (Fig. 3A4, B4, C4) differentiation could be observed.

### Expression of SP70 in different tissues

**Fig. 4:**
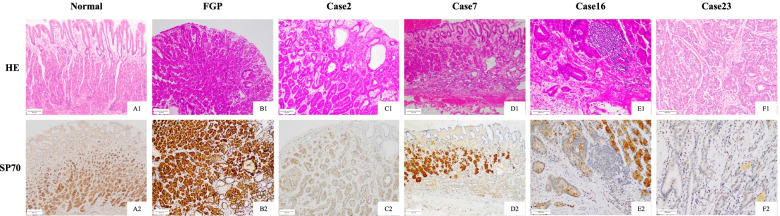
HE staining and immunostaining of SP70 in normal gastric mucosa, FGP, and different subtypes of fundic gland type neoplasm

Immunohistochemical analysis of SP70 showed diffuse and strong positivity in the normal fundic glands, but not in gastric pit cells (Fig. 4A1 and A2). A similar expression pattern could be observed in all cases of FGPs (100%). In contrast, SP70 was down-expressed in the neoplastic group (Fig. 4C–F). The positive rate of SP70 in the neoplastic group (6/23, 26.1%) was significantly lower than that in FGPs (100%) (P<0.0001). There was no difference in SP70 expression between OGAs (3/12, 25.0%) and GA-FGs (3/11, 27.2%) group (*P*>0.05).

**Fig. 5:**
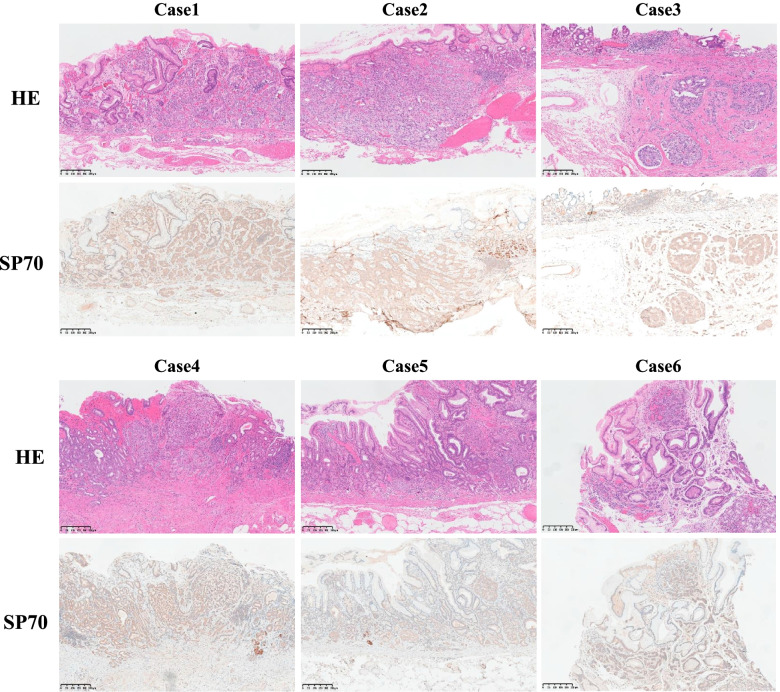
The expression of SP70 in 6 cases of NETs in the gastric body

As for these 6 cases of NETs, SP70 expression was weak to moderate intensity in the majority of tumor cells (neither like the strong expression in normal fundic glands, nor down/loss-expression in fundic gland neoplasms) (Fig. 5).

## Discussion

SP70 specifically expresses in lung cancer cells, but rare positively or negatively reacts to human small-cell lung cancer (SCLC), pulmonary pseudotumor, and other epithelial tumors [14]. It has been proven to be a key protein that can regulate tumor proliferation and apoptosis, as well as a biomarker for NSCLC [15].

SP70 has not been used as a diagnostic biomarker. In this study, we testified and compared the SP70 expression pattern in different lesions, including FGPs, OGAs, GA-FGs, and NETs. Our IHC analysis showed that the SP70 staining pattern of fundic gland neoplasms (OGAs and GA-FGs) differed from that of FGPs and NETs. Unlike diffuse and strong staining in the normal fundic glands and FGPs, as well as a weak to moderate positivity in NETs, a significant down-expression or complete loss of SP70 was observed in OGAs and GA-FGs. These findings indicated that SP70 could be used as a diagnostic biomarker with high sensitivity and specificity for the diagnosis of neoplasms with fundic gland differentiation.

SP70, as an auxiliary biomarker, can be very helpful in the diagnosis of gastric neoplasm of the fundic gland differentiation. The challenges in pathological diagnosis lie in: first, it is a relatively rare entity; second, the extremely well differentiation; and third, cytological atypia is mild, despite discernible architectural abnormities. These features easily lead to misdiagnosis in inexperienced hands, especially in biopsy specimens. Particularly, it would be more difficult to discriminate GA-FGs from less commonly dysplastic FGPs (with mild atypia) [16] and OGAs (with a confusing submucosal involvement) [1] in biopsy specimens. Current auxiliary IHC biomarkers (including pepsinogen-I, MUC6, and focal H+/K+-ATPase) could serve to confirm fundic gland differentiation, but not be able to distinguish neoplastic lesions from non-neoplastic lesions. Besides, SP70 could also be helpful in identifying neuroendocrine neoplasm and fundic gland neoplasms, in combination with the neuroendocrine markers (ChromograninA and Synaptophysin). Therefore, the clear-cut difference in the SP70 staining pattern of fundic gland neoplasms (OGAs and GA-FGs) could be an ideal biomarker to identify this neoplasm. It would be really useful in cases with atypical morphology.

The differential diagnosis of fundic gland neoplasms and non-neoplastic lesions is clinically relevant. Although most of GA-FGs appear to show an indolent behavior, a small subset of these tumors with deeper submucosal invasion could transform into poorly differentiated adenocarcinomas [5, 17, 18]. Ueyama et al. [2, 18] reported the shift in cell differentiation from a GA-FG component into high-grade adenocarcinoma with lymph node metastasis. Moreover, OGAs and GA-FGs were of similar histogenesis [4] and chief cell adenoma was considered to be a precursor to the chief cell-predominant adenocarcinoma. As a result, endoscopic resection should be performed at the time of diagnosis of neoplastic fundic glands (OGA and GA-FG), in case of further progression [11]. According to the previous study, sporadic FGPs were self-limiting neoplasms, which had little chance of transforming into carcinomas [4]. Therefore, the patients only need to undergo routine follow-up.

**Fig. 6:**
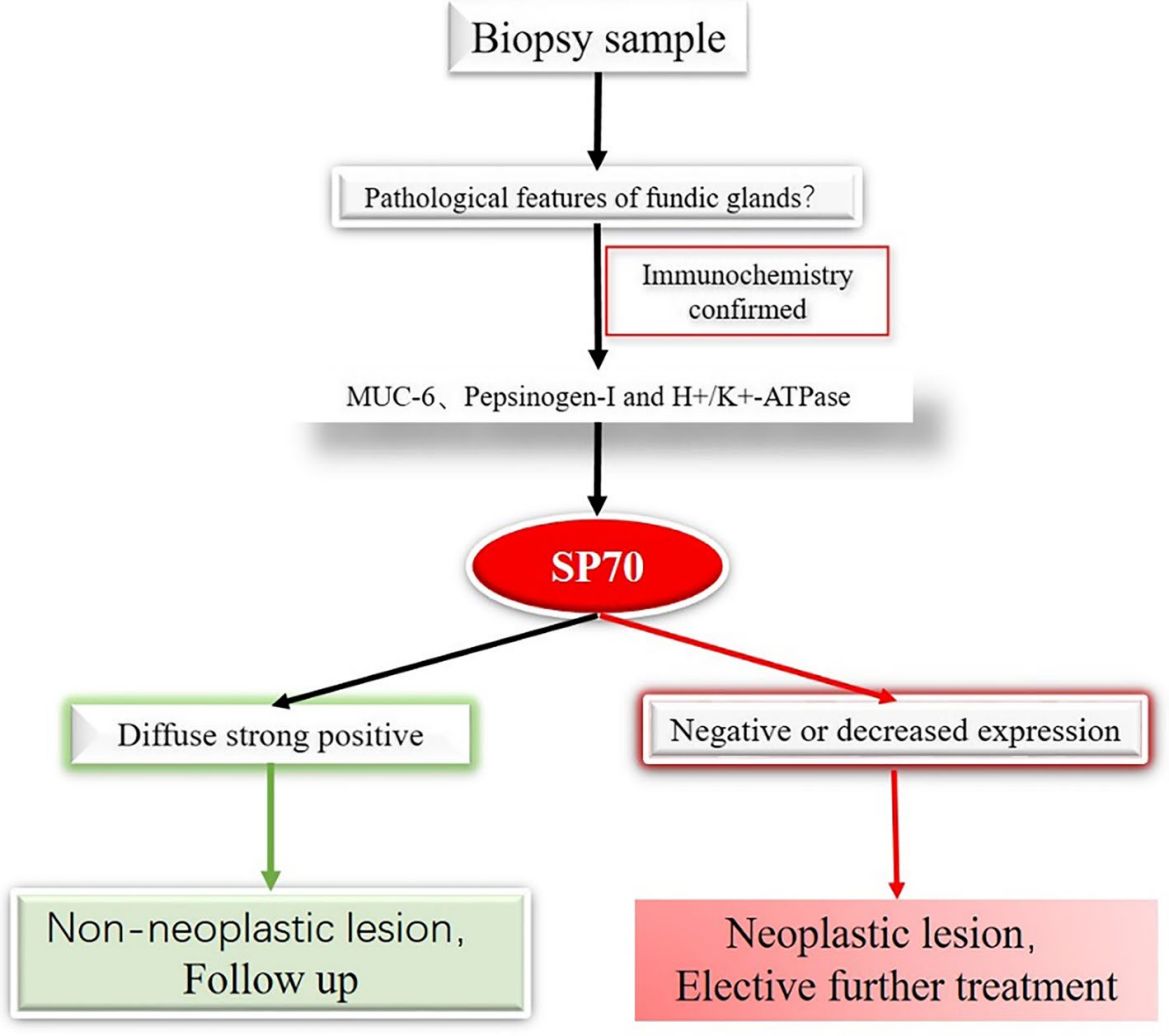
Pathways for diagnosis of fundus gland tumors based on immunohistochemistry

SP70 is the first diagnostic biomarker that can be used to distinguish gastric fundic gland neoplasms from non-neoplastic lesions. Based on the findings, we proposed a diagnosis flow chart which we believe could improve the accuracy and reliability of diagnosing the neoplasms in biopsy specimens. The specimens will be stained with currently used biomarkers including muc6, H+/K+-ATPase, and Pepsinogen-I to confirm the fundic gland differentiation. Subsequently, a diagnosis of a neoplasm or a non-neoplastic lesion can be made based on the expression of SP70 (Fig. 6).

There are several limitations. Firstly, its retrospective nature with relatively small sample number; Secondly, OGAs and GA-FGs share similar staining patterns and are not be able to distinguish by SP70. A morphological continuum exists from OGA to GA-FG, and there is an ongoing discussion on whether OGA should be regarded as an intramucosal component of GA-FG. Therefore, these two entities may share identical IHC phenotype and are unnecessary to differentiate them by IHC.

## Conclusion

In summary, it found that SP70 was strongly and diffused expressed in normal fundic glands and FGPs, while it showed a down-expression or loss of expression in fundic gland neoplasms, including OGPs and GA-FGs. Therefore, the down-expression of SP70 is a specific feature of gastric fundic gland neoplasms. The application of SP70 protein coupled with other fundic gland-associated markers could improve the diagnostic accuracy of neoplastic lesions with fundic gland differentiation. SP70 could be used as a new diagnostic biomarker to identify gastric fundic gland neoplasms.

## Data Availability

Data could be accessed on request from the authors.

## References

[1] Chan K, Brown IS, Kyle T, Lauwers GY, Kumarasinghe MP (2016). Chief cell-predominant gastric polyps: a series of 12 cases with literature review. Histopathology.

[2] Ueyama H, Yao T, Nakashima Y, Hirakawa K, Oshiro Y, Hirahashi M (2010). Gastric adenocarcinoma of fundic gland type (chief cell predominant type): proposal for a new entity of gastric adenocarcinoma. Am J Surg Pathol.

[3] Miyazawa M, Matsuda M, Yano M, Hara Y, Arihara F, Horita Y (2016). Gastric adenocarcinoma of the fundic gland (chief cell-predominant type): a review of endoscopic and clinicopathological features. World J Gastroenterol.

[4] Singhi AD, Lazenby AJ, Montgomery EA (2012). Gastric adenocarcinoma with chief cell differentiation: a proposal for reclassification as oxyntic gl and polyp/adenoma. Am J Surg Pathol.

[5] Ushiku T, Kunita A, Kuroda R, Shinozaki-Ushiku A, Yamazawa S, Tsuji Y (2020). Oxyntic gland neoplasm of the stomach: expanding the spectrum and proposal of terminology. Mod Pathol.

[6] Nagtegaal ID, Odze RD, Klimstra D, Paradis V, Rugge M, Schirmacher P (2020). Board WHOCoTE: the 2019 WHO classification of tumours of the digestive system. Histopathology.

[7] Fan X, Yang XS, Bai P, Ren YB, Zhang L, Li X (2020). Gastric adenocarcinoma of the fundic gland type: a case report. Medicine (Baltimore).

[8] Lee TI, Jang JY, Kim S, Kim JW, Chang YW, Kim YW (2015). Oxyntic gland adenoma endoscopically mimicking a gastric neuroendocrine tumor: a case report. World J Gastroenterol.

[9] Peng T, Deng L, Hou L, Wang Y, Wang R, Gao R (2020). A case report: endoscopic diagnosis and treatment of gastric adenocarcinoma of fundic gland type. J Gastrointest Cancer.

[10] Miyazawa M, Matsuda M, Yano M, Hara Y, Arihara F, Horita Y (2015). Gastric adenocarcinoma of fundic gland type: five cases treated with endoscopic resection. World J Gastroenterol.

[11] Okumura Y, Takamatsu M, Ohashi M, Yamamoto Y, Yamamoto N, Kawachi H (2018). Gastric adenocarcinoma of fundic gland type with aggressive transformation and lymph node metastasis: a case report. J Gastric Cancer.

[12] Ishibashi F, Fukushima K, Ito T, Kobayashi K, Tanaka R, Onizuka R (2019). Influence of helicobacter pylori infection on endoscopic findings of gastric adenocarcinoma of the fundic gland type. J Gastric Cancer.

[13] Gu C, Luo Y, Zhang S, Xu J, Zhang J, Ju H (2020). MAb NJ001 inhibits lung adenocarcinoma invasiveness by directly regulating TIMP-3 promoter activity v ia FOXP1 binding sites. Thorac Cancer.

[14] Pan S, Wang F, Huang P, Xu T, Zhang L, Xu J (2012). The study on newly developed McAb NJ001 specific to non-small cell lung cancer and its biological cha racteristics. PLoS One.

[15] Xu J, Zhang S, Zhang W, Xie E, Gu M, Wang Y (2020). SP70-targeted imaging for the early detection of lung adenocarcinoma. Sci Rep.

[16] Tohda G, Osawa T, Asada Y, Dochin M, Terahata S (2016). Gastric adenocarcinoma of fundic gland type: endoscopic and clinicopathological features. World J Gastrointest Endosc.

[17] Benedict MA, Lauwers GY, Jain D (2018). Gastric adenocarcinoma of the fundic gland type: update and literature review. Am J Clin Pathol.

[18] Ueo T, Yonemasu H, Ishida T (2014). Gastric adenocarcinoma of fundic gland type with unusual behavior. Dig Endosc.

